# QTL Analysis Reveals Conserved and Differential Genetic Regulation of Maize Lateral Angles above the Ear

**DOI:** 10.3390/plants12030680

**Published:** 2023-02-03

**Authors:** Yanbin Zhu, Bo Song, Yanling Guo, Baobao Wang, Changcheng Xu, Hongyu Zhu, Lizhu E, Jinsheng Lai, Weibin Song, Haiming Zhao

**Affiliations:** 1 State Key Laboratory of Plant Physiology and Biochemistry, National Maize Improvement Center, Department of Plant Genetics and Breeding, China Agricultural University, Beijing 100193, China; 2 National Key Laboratory of Maize Biological Breeding, Key Laboratory of Genetics and Breeding of Main Crops in Northeast Region, Ministry of Agriculture and Rural Affairs, Liaoning Dongya Seed Industry Co., Ltd., Shenyang 110164, China; 3 Sanya Institute of Henan University, Sanya 572025, China; 4 Biotechnology Research Institute, Chinese Academy of Agricultural Sciences, Beijing 100081, China

**Keywords:** maize, leaf angle (LA), tassel branch angle (TBA), quantitative trait locus (QTL)

## Abstract

Improving the density tolerance and planting density has great importance for increasing maize production. The key to promoting high density planting is breeding maize with a compact canopy architecture, which is mainly influenced by the angles of the leaves and tassel branches above the ear. It is still unclear whether the leaf angles of different stem nodes and tassel branches are controlled by similar genetic regulatory mechanisms, which limits the ability to breed for density-tolerant maize. Here, we developed a population with 571 double haploid lines derived from inbred lines, PHBA6 and Chang7-2, showing significant differences in canopy architecture. Phenotypic and QTL analyses revealed that the genetic regulation mechanism was largely similar for closely adjacent leaves above the ears. In contrast, the regulation mechanisms specifying the angles of distant leaves and the angles of leaves vs. tassel branches are largely different. The *liguless1* gene was identified as a candidate gene for QTLs co-regulating the angles of different leaves and the tassel branch, consistent with its known roles in regulating plant architecture. Our findings can be used to develop strategies for the improvement of leaf and tassel architecture through the introduction of trait-specific or pleiotropic genes, thus benefiting the breeding of maize with increased density tolerance in the future.

## 1. Introduction

Maize is one of the most important crops, serving as a source of food, feed and industrial materials. Maintaining a sufficient supply of maize is vital for ensuring food security worldwide. Research has shown that during the history of maize breeding, increasing planting density and density tolerance has been the most important technical measure to improve maize yield. For example, Duvick conducted comprehensive studies on the hybrids released in the United States from 1934 to 2004 and found that the yield per plant and hybrid heterosis did not largely increase during the process of increasing the yield per unit area of American corn, while the planting density and density tolerance continued to increase [1,2,3]. Mansfield et al. also found that the increase in corn yield in the United States from 1930 to 2010 was strongly correlated with the planting density and variety density tolerance [4]. These observations demonstrate that increasing planting density and cultivating varieties with density tolerance are important for increasing maize production.

A key measure to increase density tolerance in maize is to breed plants with a compact architecture, which is conducive to increasing ventilation and light transmission and reducing the competition between plants. For example, introgressing the favorable allele of the leaf angle-regulating gene *Upright Plant Architecture2* into modern hybrids was shown to significantly enhance maize yields under high-density conditions [5]. It has been reported that the most important requirement for maize ideotype is the compact plant configuration above the ear [6]. The three leaves around the upper-most ear (the first leaf above uppermost ear, the leaf of the uppermost ear and the first leaf below the uppermost ear) are vital to the formation of maize yield. The compact upper ear configuration is vital for ventilation, light transmission, the interception of light energy, and maintenance of effective photosynthetic efficiency. During the process of modern maize breeding, the leaves above the ear became more and more compact [7,8].

The canopy of corn plants is generally composed of tassels and three to nine leaves above the ear [8]. The angles of leaves and tassels are the keys to determining the light transmittance and canopy structure [9,10]. Therefore, it is very important to analyze the genetic basis of regulation of these angles and to shape a compact canopy structure for cultivating density-tolerant maize varieties. However, it is still unclear whether the leaf angles of different nodes and tassel branch angles are regulated differently, which limits our ability to breed density-tolerant plants.

In this study, we investigated the angles of the first (LA1), second (LA2), third (LA3), and flag leaves (FLA) above the top-most ear, and the angle of the first tassel branch (TBA) using a double haploid (DH) population derived from two inbred lines, PHBA6 and Chang7-2, with significant differences in leaf angle and tassel branch angle. QTL analysis of these traits was carried out in the DH population using a high-density linkage map. We found that the genetic regulatory basis was largely similar between adjacent leaves but relatively different between leaves and tassel branches. The *liguless1* (*LG1*) gene was identified as a candidate gene for QTLs controlling leaf and tassel branch angles, and it may play an important role in the regulation of these traits. These results are important for guiding the targeted or universal improvement of specific leaf or tassel configurations in the future.

## 2. Results

### 2.1. Phenotyping of a DH Population Derived from PHBA6 × Chang7-2

**Figure 1:**
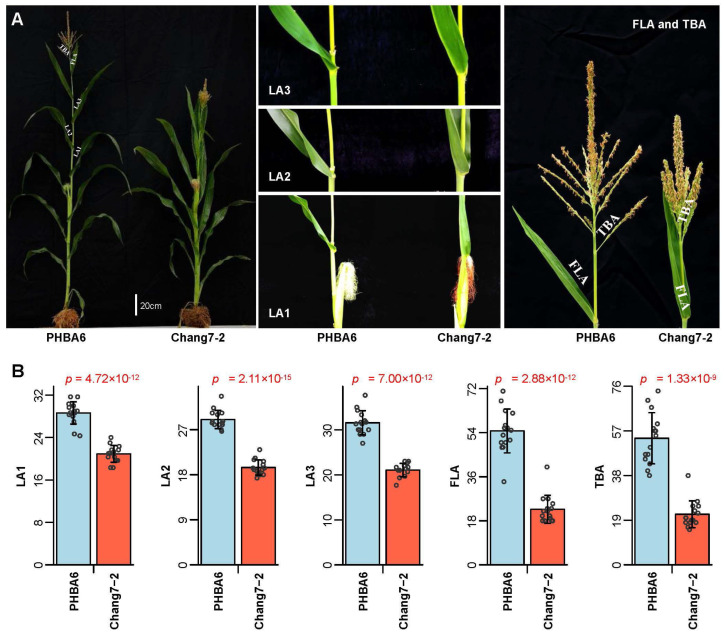
Phenotypic differences between the parental inbred lines PHBA6 and Chang7-2. (**A**) Comparison of plant morphology and angles of the first leaves (LA1), second leaves (LA2), third leaves (LA3), and flag leaves (FLA) above the upmost ear and the angles of the first tassel branch (TBA) between PHBA6 and Chang7-2. (**B**) Bar plot of LA1, LA2, LA3, and FLA above the upmost ear and the TBA of PHBA6 (n = 15) and Chang7-2 (n = 15). *p*-values of two tailed *t*-test are shown above each plot.

To study the genetic basis for regulation of lateral angles above the ear, we selected two inbred lines, PHBA6 and Chang7-2, with significant differences in leaf and tassel angles and constructed a DH population composed of 571 lines. Compared with Chang7-2, PHBA6 had a larger leaf and tassel branch angles (Figure 1).

**Figure 2:**
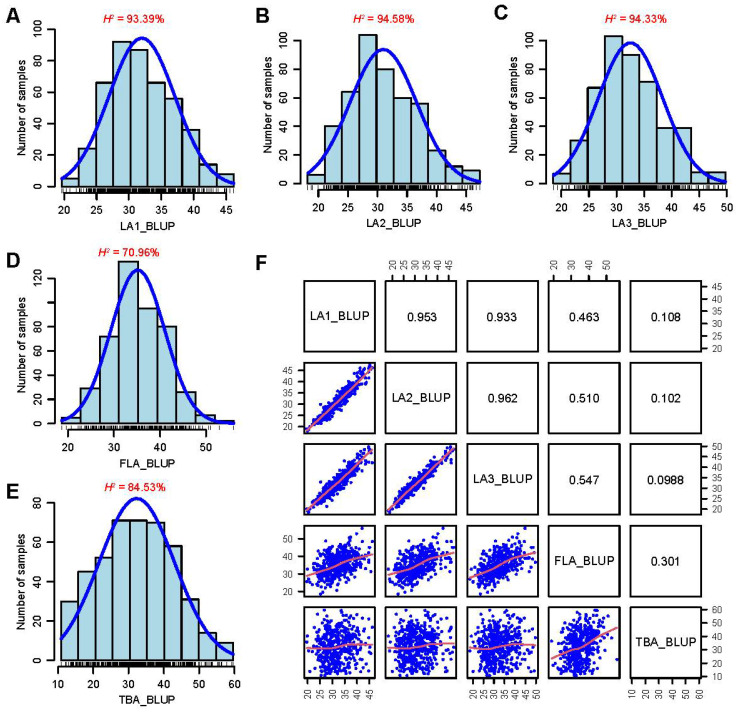
The distributions of leaf and tassel angles and correlation between traits in the 571 DH lines. (**A**–**E**) The phenotypic distributions and heritabilities of LA1 (**A**), LA2 (**B**), LA3 (**C**), FLA (**D**), and TBA (**E**) of the 571 DH lines. *H^2^*, heritability. (**F**) Correlation among LA1, LA2, LA3, FLA, and TBA of the 571 DH lines. The correlation coefficients are shown above the diagonal and scatterplots are shown below the datapoints.

We measured the LA1, LA2, LA3, and FLA above the top-most ear and the TBA of PHBA6, Chang7-2 and 571 DH lines in six environments and calculated the best linear unbiased prediction (BLUP) values (Supplementary Table S1). The mean of LA1, LA2, LA3, FLA and TBA for PHBA6 are 28.6, 29.0, 31.6, 54.7 and 53.9, respectively. The means of LA1, LA2, LA3, FLA and TBA for Chang7-2 are 20.9, 19.4, 21.0, 22.7 and 21.5, respectively. The means of LA1, LA2, LA3, FLA and TBA for the progenies of 571 DH lines are 32.0, 31.0, 32.5, 35.2 and 32.3, respectively. The LA1 of 571 DH lines varied from 19.6 to 46.1 degrees. The LA2 of 571 DH lines varied from 18.2 to 47.3 degrees. The LA3 of 571 DH lines varied from 18.6 to 49.7 degrees. The FLA of 571 DH lines varied from 18.6 to 56.0 degrees. The TBA of 571 DH lines varied from 10.8 to 59.6 degrees. All of these five traits showed transgressive segregation. Among them, the FLA and TBA showed wider variability. We also found that LA1, LA2, and LA3 had higher broad-sense heritabilities (*H^2^* ≥ 93.39%) than TBA (*H^2^* = 84.53%) and FLA (*H^2^* = 70.96%) (Figure 2). Almost all the lateral angle-related traits showed a continuous normal distribution, indicating that these traits are controlled by quantitative trait loci (QTLs), which is similar to the conclusions reported previously [11,12,13].

Correlation analysis of BLUP values for different traits showed a very strong correlation between the angles of adjacent leaves (LA1, LA2, and LA3, *r* ≥ 0.933). The correlation between angles of leaves at farther distances apart were still significant; however, the correlation gradually decreased with the distance between the position of different leaves. For example, the correlation coefficient between FLA and LA3 was 0.547, whereas that between FLA and LA1 was only 0.463. The TBA and FLA, which are adjacent to each other, were moderately correlated, whereas the correlations of TBA with LA1, LA2, and LA3 were weak (Figure 2). The significant correlation among these traits indicated that there may be some shared genetic regulators of these angles, while the difference in correlation also indicated that some regulators may be unique or may differentially regulate these angles [14].

### 2.2. High-Density Linkage Map Construction

We next analyzed the genetic basis of lateral angle regulation using the massively parallel 3’ end RNA sequencing (MP3RNA-seq) strategy [15] to genotype the 571 DH lines. After SNP calling and strict filtering (see Materials and Methods), we retained 436 DH lines (135 lines with missing genotype data were excluded from the analysis) and 26,917 non-redundant SNPs for high density linkage map construction. The total length of the genetic map was 833.94 cM, and the average genetic distance and physical distance between the markers were 0.033 cM and 0.082 Mb, respectively (Table 1), indicating the high resolution of our map.

**Figure 3:**
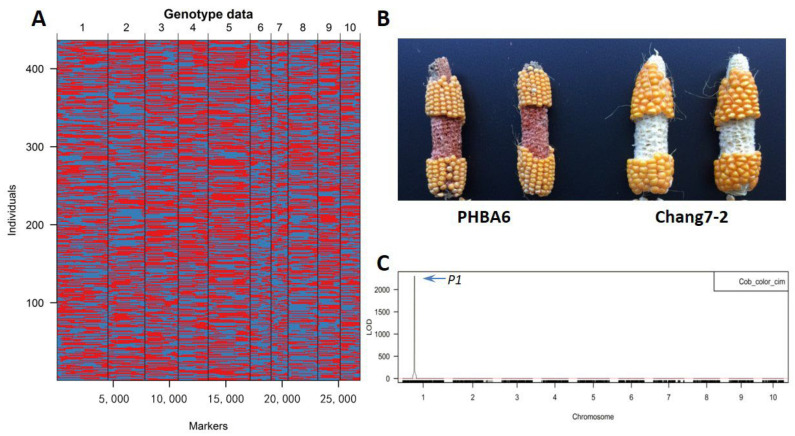
A genetic map of the DH population and QTL mapping of the cob color gene *Pericarp1* (*P1*). (**A**) Graphical representation of the genotypes of 571 DHs. Red, PHBA6 genotype; blue, Chang7-2 genotype. (**B**) Image showing the cob colors of PHBA6 (red) and Chang7-2 (white). (**C**) Results of QTL mapping of cob color in the DH population. The QTL peak corresponding to the *P1* gene is indicated. The dotted red line corresponds to the cutoff line.

To verify the accuracy and validity of our map, we first performed QTL analysis of cob color with simple genetic structure in the DH population. The difference in cob color between the parents of the DH population was remarkable, so the cob color traits of 311 lines in the DH population were successfully scored. The ratio of red cobs to white cobs was 141:170, which very close to the 1:1 ratio expected if cob color is controlled by a single gene (Chi-square test, *p* = 0.100) [16]. QTL analysis revealed a very significant QTL locus on chromosome 1 between 47.589118 and 48.956707 Mb (peak at 48.336939 Mb, LOD = 2308.5), which is located within the tandem repeat region containing the known cob color gene *Pericarp color1* (*P1*) [17] (Figure 3). The precise mapping of the *P1* gene demonstrated the quality of our map.

### 2.3. QTL Analysis of Lateral Angles in the DH Population

To clarify the genetic basis for control of lateral angles above the top-most ear, QTL mapping was performed for the five lateral angle traits in the DH population using the high-density linkage map (Table 1). A total of 42 lateral angle-related QTLs were identified (Table 2), with 12, 8, 9, 6, and 7 QTLs identified for LA1, LA2, LA3, FLA, and TBA, respectively. The additive effects of these QTLs varied from 0.82° to 7.57° with an average of 3.03°. For most QTLs, the allele decreasing the angle was contributed by Chang7-2, which indicates that Chang7-2 may be an important donor for the improvement of compact plant architecture above the ear. The phenotypic variation explained by these QTLs ranged from 0.85% to 21.8%, with an average of 6.42%. Most of the lateral angle QTLs (35/42) were minor effect QTLs, which was consistent with the findings from previous genetic analyses of leaf angle [8,11,12,13] and also reflected the complexity of the regulation of the angle traits. The detected QTLs explained 64.44%, 57.15%, 52.57%, 44.33%, and 50.90% of the phenotypic variation for LA1, LA2, LA3, FLA, and TBA, respectively. These results were consistent with the findings from phenotypic analysis that LA1 has the highest heritability and FLA has the lowest (Figure 2).

We next analyzed the distribution of the 42 QTLs and the overlap between them. QTLs were found on all chromosomes except for chromosomes 6 and 7. There were seven overlapping QTLs among LA1, LA2, and LA3 (Table 3), and these QTLs explained a large portion of the phenotypic variation of these three traits (34.21–48.32%), indicating that the mechanisms regulating these different lateral angles are very similar. Interestingly, we found that the overlapping QTLs between adjacent leaf angles (LA1 vs. LA2, LA2 vs. LA3) could explain more phenotypic variation than those between more distant angles (LA1 vs. LA3). There were no overlapping QTLs between the most distant leaf angles (FLA vs. LA3). For FLA vs. LA1 and FLA vs. LA2, there was only one overlapping QTL each, and the proportion of explained phenotypic variation was small (12.11–21.08%). These results implied that the regulation mechanisms for the angles of distant leaves may be largely different. We further investigated the overlapping QTLs between the tassel and leaf angles. The result indicated that there was only one overlapping QTL between LA1, LA2, FLA and TBA, respectively. No overlapping QTL was detected between TBA and LA3. These results indicated that although there may be some similar factors regulating the angle of tassel and leaf, their genetic basis was generally largely different.

### 2.4. Candidate Gene Analysis

**Figure 4:**
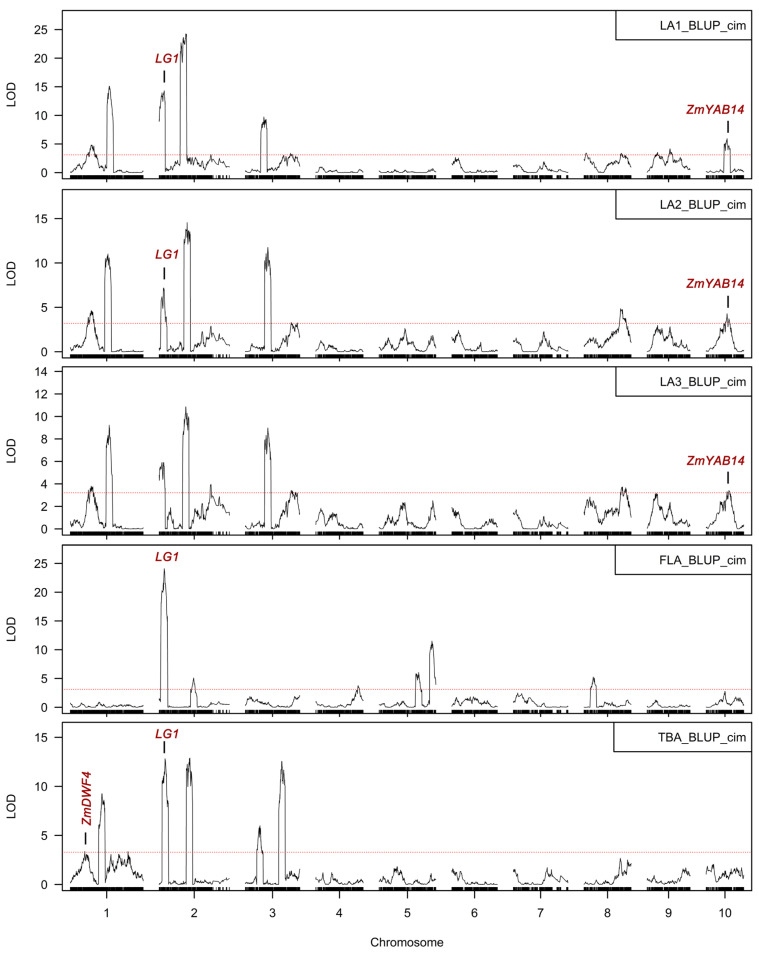
Results of QTL mapping of LA1, LA2, LA3, FLA, and TBA. The *y*-axis represents -log10 transformed p-values from *t*-testing; the red dotted line represents the cutoff. The positions of the candidate genes *LG1*, *ZmDWF4*, and *ZmYAB14* are indicated by vertical bold lines. LA1_BLUP_cim, LA2_BLUP_cim, LA3_BLUP_cim, FLA_BLUP_cim and TBA_BLUP_cim represents the QTL mapping that was conducted with the BLUP values calculated for LA1, LA2, LA3, FLA, and TBA across different environments separately and the composite interval mapping was used mapping each trait.

There have been many studies on the genetic basis of maize leaf angle and tassel branch angle, and genes affecting the lateral angles have been reported. When searching our QTL intervals, we found several previously reported functional genes or homologs of these genes that may be candidate genes (Figure 4). The same intervals on chromosome 10 for *qLA1_10*, *qLA2_10*, and *qLA3_10* contained a YABBY-like transcription factor gene, *ZmYAB14* (*Zm00001d025944*). Previous reports have shown that the YABBY family of transcriptional regulators regulate the angle and architecture of maize leaves [18]. Therefore, *ZmYAB14* is a likely candidate gene for these QTLs. *qTBA_1a*, which is located at the beginning of chromosome 1 contains the important maize brassinosteroid (BR) synthesis gene *ZmDWF4* (181 kb from the QTL peak). *ZmDWF4* encodes a cytochrome P450, steroid 22-alpha-hydroxylase, that catalyzes C-22 hydroxylation in the BR biosynthesis pathway [19]. BR plays an important role in the regulation of plant lateral angles. Thus, *ZmDWF4* may be a candidate gene for *qTBA_1a*. Finally, the key lateral boundary definition gene *LG1*, which encodes a square promoter binding transcriptional regulator [20], was located in the QTL confidence intervals of *qLA1_ 2a*, *qLA2_2a*, *qFLA_2a*, and *qTBA_2A*, very close to the peaks (43, 74, 45, and 573 kb, respectively). This, combined with the known role of *LG1* in regulating LA and TBA [21,22], makes *LG1* a likely candidate gene for these QTLs controlling LA and TBA.

## 3. Discussion

### 3.1. A Large-Scale DH Line Population Improving the Accuracy of QTL Mapping with BLUPs

Previous studies have mapped many QTLs for lateral angles in maize using F2:3 populations, recombinant inbred line (RIL) populations or a few other segregating populations [23,24,25,26,27]. Compared with RIL populations, the DH approach quickly converts heterozygous materials to completely homozygous lines, that greatly reduces the time to build a stable mapping population [28]. There is no dominant effect of genes in the DH lines; thus, the additive and epistatic effects of quantitative trait genes can be studied more accurately. Moreover, the DH populations can be used to eliminate the influence of competition among individual plants and reduce the environmental test error compared with the F2:3 populations and the other early segregating populations. The cost of sequencing continues to decrease along with the development of high-throughput sequencing technology. Meanwhile the system for the rapid creation of large-scale DH lines has been established gradually [29,30,31]. Therefore, DH populations are ideal for mapping QTLs and identifying candidate genes [32,33].

**Figure 5:**
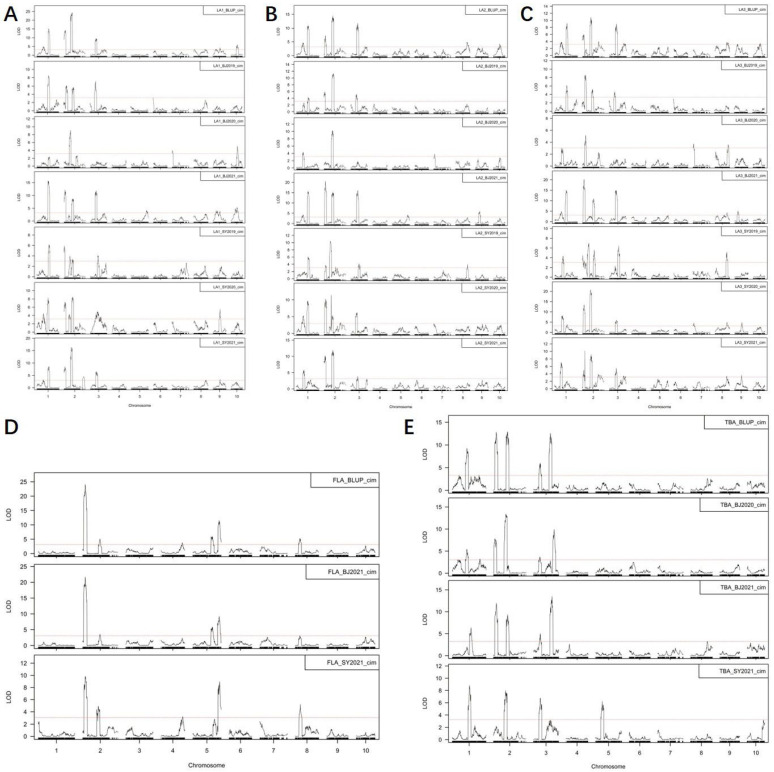
Comparisons between QTLs for lateral angles of BLUPs and individual environments. (**A**–**E**) The results of QTL mapping for LA1 (**A**), LA2 (**B**), LA3 (**C**), FLA (**D**) and TBA (**E**) of BLUP and individual environments, separately. The *y*-axis represents -log10 transformed p-values from *t*-testing; the red dotted line represents the cutoff. LA1_BLUP_cim, LA2_BLUP_cim, LA3_BLUP_cim, FLA_BLUP_cim and TBA_BLUP_cim represents the QTL mapping that was conducted with the BLUP values calculated for LA1, LA2, LA3, FLA, and TBA across different environments separately and the composite interval mapping was used mapping each trait. BJ2019, BJ2020, BJ2021, SY2019, SY2020 and SY2021 represents the environment of the Haidian district in Beijing in 2019 and 2020, and Shunyi district in Beijing in 2021 and the Shenbei district in Shenyang in 2019, 2020 and 2021.

BLUPs could minimize the phenotyping errors and best estimate the genetic effect influenced for a trait. BLUPs, instead of values of individual environments, were widely used in QTL mapping or GWAS study [13,34,35,36]. Thus, analysis by BLUP is more suitable for our purpose, revealing the conserved and differential genetic regulation of maize leaf and tassel branch angles. In fact, we also performed QTL analysis for individual environments. We summarized the QTL results for individual environments as following figures (Figure 5). As shown in the figures, most of the QTLs detected by BLUP were stably detected by individual environments. In this study, we used the BLUP values of 571 DH lines with 26,917 SNPs to identify 42 QTLs for lateral angle traits. The phenotypic variation explained by these QTLs ranged from 0.85% to 21.8%, with an average of 6.42%. The confidence intervals of some of these QTLs were less than 1 Mb in length. This demonstrates the mapping accuracy that can be obtained when using large-scale maize DH line populations.

### 3.2. Comparison with Previous QTL Studies

We compared the published QTL/genes for lateral angle with those identified in this study. The most prominent region, *qLA1_2a*, *qLA2_2a* and *qFLA_2a* for LA were found to be located nearby the known classic *LG**1*** gene on chromosome 2. The *LG1* gene encodes a squamosa promoter binding (SPB) translational regulator, which plays a key role in the process of ligule and auricle formation [20,37]. Tian et al. detected a significant QTL for leaf angle in the 2-Mb region at the nearby *LG1* on chromosome 2 in the maize nested association mapping (NAM) population [13]. Ku et al. used a meta-QTL analysis to find an mQTL in the region between umc1165 and bnlg1297, which overlapped with the location of *LG1* in F2:3 families of Yu823 and Yu87-1 [12]. Li et al. identified a QTL region (*qLA2a*) that ranged from 3.09 to 4.30 Mb on chromosome 2, which was located nearby to the interval of the *LG1*, using three RIL populations derived from crossing Huangzaosi with Huobai, Weifeng322, and Lv28 [25]. Ding et al. and Tang et al. detected *qLA2-1* and *qLA2.1* in the same region, bin 2.01 on chromosome 2, which overlapped with the location of *LG1*, in a four-way cross population of D276, D72, A188 and Jiao51 and a RIL population derived from B73 and SICAU1212, separately [23,38]. These QTLs that were detected in different genetic backgrounds and environments shared a high congruence, which supported the candidacy of *LG1* for *qLA1_2a*, *qLA2_2a* and *qFLA_2a*. In addition to lacking ligules and auricles, *lg1* mutants have significantly smaller leaf and tassel branch angles [21,22], which is consistent with this gene being located in QTL intervals for both LA and TBA (*qLA1_2a*, *qLA2_2a*, *qFLA_2a*, and *qTBA_2A*). However, the leaves of *lg1* mutants is lacking a proper ligular region and displays an upright habit of growth; both PHBA6 and Chang7-2 have normal ligules and auricles. We speculate that functional variants may be located in the regulatory region of *LG1*. This locus, as the only one identified, may regulate almost all lateral angles (LA1, LA2, FLA, TBA) above the ear. In the future, this locus can be used to improve the ear configuration of breeding materials through molecular breeding. Further exploration of the functional natural variation of this locus would be of great benefit to breeding of density tolerant maize in the future. *qLA1_10*, *qLA2_10*, and *qLA3_10* were in the same interval on chromosome 10 which contained a YABBY-like transcription factor gene, *ZmYAB14*. The YABBY family of transcriptional regulators regulate the angle and architecture of maize leaves [18]. This QTL region was closely neighboring the QTLs for lateral angles identified on chromosome 10 in the previous studies [13,25,26,39,40]. There was no known gene co-location with another large-effect QTL *qLA2_3a* and its overlapping QTL *qLA3_3a*, suggesting that there was an undiscovered gene controlling maize leaf angle.

*qTBA_1a*, which is located at the beginning of chromosome 1 contains the important maize brassinosteroid (BR) synthesis gene *ZmDWF4* (181 kb from the QTL peak). Ku et al. detected the corresponding QTL region for LA (bin 1.02 of chromosome 1) where the *DWAF4* gene was located using two different F2:3 populations. Liu et al. detected the *ZmDWF4* gene co-locating with one large-effect QTL, *qLA1_2* for LA by using high-density SNP markers and a F2:3 population of H082183 × Lv28. Dzievit et al. identified the *ZmDWF4* gene in one prominent genomic bin on chromosome 1 for LA across the F2 and F2:3 generation for the B73 and Mo17 populations. Therefore, *ZmDWF4* may be a candidate gene for *qTBA_1a*. *ZmDWF4* and encodes a cytochrome P450, steroid 22-alpha-hydroxylase, that catalyzes C-22 hydroxylation in the BR biosynthesis pathway [19]. BR plays an important role in the regulation of plant lateral angles. Previous studies have revealed the effects of plant phytohormones, especially BR, in regulating leaf angles. Zhao et al. expounded that *LC2* may regulate leaf angle through a BR-independent pathway and participate in the feedback control of BR signaling, using a rice leaf inclination2 (*lc2*, three alleles) mutant [41]. Feng et al. confirmed that *SLG*, a BAHD acyltransferase-like protein gene is involved in BR homeostasis by positively regulating endogenous BR levels to control leaf angle for planting density in rice [42]. Liu et al. found that *TaSPL8* might regulate the lamina joint, the tissue connecting the leaf blade and sheath by binding to the promoter of the brassinosteroid biogenesis gene CYP90D2, and activating its expression [43]. We believe that the causal genes for our angle QTLs may also be involved in BR synthesis/signaling, and this needs to be further studied in the future.

## 4. Materials and Methods

### 4.1. Plant Materials and Phenotypic Data Collection

For DH lines production, the F1s, derived by crossing of PHBA6 and Chang7-2, were crossed with a maize haploid inducer CAU3 in 2014 in Sanya (The F1s were used as female parent, and CAU3 as male parent). The putative haploid kernels were selected based on the lack of *R1*-mediated purple anthocyanin in the scutellum of the haploid embryo [29] and planted at the field nursery during the 2015 summer in Beijing. The putative haploids were further screened using the features of shorter stature and smaller biomass in the field. Approximately 1% of the haploid plants were successfully self-crossed by the natural doubling method.

The DH population used here includes 571 lines, and was derived by crossing PHBA6 and Chang7-2. The DH lines and their parents were phenotyped in six environments (each location in an individual year was considered an environment), namely the Haidian (40.14° N, 116.19° E) district in Beijing in 2019 and 2020, and Shunyi (40.23° N, 116.56° E) district in Beijing in 2021 and the Shenbei (42.03° N, 123.59° E) district in Shenyang in 2019, 2020 and 2021. A randomized complete block design was used with two replications in each environment. Trials were performed with one-row plots: row length of 1 m; five plants per plot; row spacing of 0.5 m for Haidian and Shunyi districts in Beijing (80,000 plants per hectare) and row spacing of 0.6 m in the Shenbei district in Shenyang (66,666 plants per hectare). PHBA6 and Chang7-2 were used as controls, and they were planted in every 20 DH lines, alternately. All the materials were grown under natural field conditions in each environment and field management was in accordance with local practices. LA1, LA2, LA3, and FLA were measured on three plants per plot, while cob color and TBA were measured on three to five plants per plot. LA1, LA2, and LA3 measurements were collected at Haidian, Shunyi, and Shenbei. Cob color, FLA, and TBA were collected at Shunyi and Shenbei only. Cob color was divided into red and white. LA1, LA2, and LA3 were recorded as the angle between the midrib and upper stem of the first, second, third leaves above top-most ear node, respectively. FLA was recorded as the angle between midrib and upper stem of the flag leaf. TBA was recorded as the angle between the first primary branch and the central spike of tassel. BLUP values were calculated for each phenotype across different environments using the lme4 package [17] in R, and these values were used for subsequent analysis. The heritability (*H^2^*) estimates were calculated by R software as reported previously [17].

### 4.2. RNA Isolation, MP3RNA-Seq, and SNP Calling

Leaf tissues from three seedlings per DH line were bulked for total RNA extraction, which was used for construction and sequencing of cDNA libraries using the MP3RNA-seq method [15]. The DH lines for which library construction was unsuccessful, or little sequencing data were obtained (135 lines in total), were excluded from further analysis.

SNP calling was conducted using the software samtools (v0.1.16) and bcftools (v0.1.16). Only uniformly mapped reads and non-duplicated reads were used after filtering [44]. Then, the MP3RNA-seq data for PHBA6 and Chang 7-2 were analyzed for SNP identification (location: refer to the B73 genome of version 4). A total of 35,836 high-quality SNPs were detected between PHBA6 and Chang 7-2 with a read depth ≥ 5. In addition, the SNPs with partial segregation greater than 2/1 (Chi square test, *p* < 1.0 × 10^−7^) or a heterozygosity rate greater than 15% were discarded, and the DH lines with a heterozygosity rate greater than 15% were also eliminated. Ultimately, 26,917 SNPs in 571 DH lines were retained for further genetic map construction.

### 4.3. Genetic Map Construction

Genotype calling and recombination breakpoint determination for each DH were conducted using a sliding window approach [45,46] with minor modifications.

(1) In each 15-SNP window, the DH genotype was defined by the ratio of alleles from PHBA6 and Chang7-2:

The window was called homozygous if > 11/15 of the sites in the window were alleles from either of the parents or heterozygous otherwise. Using the sliding window method, we found that recombination breakpoints were detectable as a region of several heterozygous SNPs that did not exceed more than six continuous windows. Therefore, we set the heterozygous regions spanning less than seven uninterrupted windows as breakpoints and divided them into two at the midpoint. Then adjacent windows with the same genotype were merged together as a block.

(2) Blocks with fewer than five sequenced SNPs or a physical length less than 300 kb were set as missing to avoid calling false double crossover events.

(3) Adjacent windows and successive small blocks with frequently transient genotypes were merged into a larger heterozygous block, and heterozygous blocks with fewer than 15 SNPs or a physical length less than 1 Mb were set as missing to avoid false estimation.

### 4.4. QTL Analysis

The QTL analyses of lateral angles were conducted using the composite interval mapping (cim) method in the R package R/qtl as reported [46]. The LOD threshold was defined by 1000 permutations at a significance level of *p* < 0.05. The 1.5 LOD-drop method was used for defining the QTL confidence interval. A linear QTL model was used for evaluation of QTL effect size.

## Figures and Tables

**Table 1:** Profile of the linkage map.

Chr	Physical Distance (Mb)			Genetic Distance (cM)		
	Number ^a^	Mean ^b^	Max ^c^	Length ^d^	Mean ^b^	Max ^c^
1	4529	0.068	15.603	114.39	0.025	1.174
2	3276	0.075	10.891	110.28	0.034	4.788
3	2954	0.080	8.111	86.10	0.029	1.782
4	2661	0.093	17.568	74.45	0.028	2.532
5	3730	0.060	4.081	89.08	0.024	1.782
6	1864	0.093	12.173	71.68	0.038	2.660
7	1500	0.121	13.779	85.96	0.057	8.851
8	2644	0.068	5.863	74.29	0.028	1.659
9	1986	0.080	13.308	67.94	0.034	2.030
10	1773	0.085	7.159	59.77	0.033	2.532
Total	26917	0.082	17.568	833.94	0.033	8.851

^a^ The number of SNPs. ^b^ The average physical (Mb) or genetic (cM) distance between adjacent SNPs. ^c^ The maximum physical (Mb) or genetic (cM) distance between adjacent SNPs. ^d^ The total genetic distance of the chromosome.

**Table 2:** Summary of the QTLs for lateral angles identified in this study.

QTL	Overlapping QTLs	Trait	Chr	Peak Marker	Peak Position (cM)	Peak Position (Mb)	LOD	Left Position (cM)	Right Position (cM)	Left Position (Mb)	Right Position (Mb)	Var (%) ^a^	Add ^b^
*qLA1_1a*	*qLA2_1a, qLA3_1a*	LA1	1	M1_1166	32.795	48.337	4.83	30.017	37.881	43.581	59.421	5.10	1.58
*qLA1_1b*	*qLA2_1b, qLA3_1b*	LA1	1	M1_2435	61.082	198.272	15.13	59.686	63.862	197.395	201.738	11.15	2.88
*qLA1_2a*	*qLA2_2a, qFLA_2a, qTBA_2a*	LA1	2	M2_233	8.021	4.278	14.32	5.830	9.181	3.550	4.643	12.11	3.45
*qLA1_2b*	*qLA2_2b, qLA3_2b*	LA1	2	M2_1194	41.982	56.798	24.25	40.358	43.482	49.336	63.033	12.31	3.85
*qLA1_2c*	*qLA3_2c*	LA1	2	M2_3360	81.294	239.246	3.15	77.133	89.575	232.638	240.608	2.49	1.14
*qLA1_3a*		LA1	3	M3_1035	29.584	112.427	9.74	27.168	30.504	28.624	124.132	8.57	−2.63
*qLA1_3b*	*qLA2_3b, qLA3_3b*	LA1	3	M3_2745	71.527	221.433	3.38	56.828	83.324	199.396	232.901	1.14	−0.82
*qLA1_8a*		LA1	8	M8_92	4.033	2.674	3.42	0.329	10.579	0.329	5.567	1.20	1.35
*qLA1_8b*	*qLA2_8, qLA3_8*	LA1	8	M8_2408	58.637	172.527	3.36	55.855	69.779	170.455	177.712	2.96	1.71
*qLA1_9a*		LA1	9	M9_204	16.823	12.891	3.53	9.518	26.843	8.655	23.397	1.98	−0.99
*qLA1_9b*		LA1	9	M9_1140	36.426	122.524	4.17	33.416	40.704	116.939	136.756	1.89	−1.28
*qLA1_10*	*qLA2_10, qLA3_10*	LA1	10	M10_1122	33.420	125.573	5.94	30.773	34.804	110.756	129.677	3.55	−1.96
*qLA2_1a*	*qLA1_1a, qLA3_1a*	LA2	1	M1_1171	33.141	48.957	4.65	29.211	37.881	40.599	59.421	6.33	1.82
*qLA2_1b*	*qLA1_1b, qLA3_1b*	LA2	1	M1_2419	59.108	197.050	11.03	54.252	61.888	184.642	199.718	7.80	2.73
*qLA2_2a*	*qLA1_2a, qFLA_2a, qTBA_2a*	LA2	2	M2_224	7.328	4.157	7.19	6.407	8.601	3.763	4.552	14.18	4.26
*qLA2_2b*	*qLA1_2b, qLA3_2b*	LA2	2	M2_1366	43.942	92.168	14.57	41.174	45.227	50.706	103.717	12.12	4.38
*qLA2_3a*	*qLA3_3a*	LA2	3	M3_1499	35.920	160.130	11.77	34.999	36.958	157.122	162.370	9.14	−3.20
*qLA2_3b*	*qLA1_3b, qLA3_3b*	LA2	3	M3_2764	72.450	221.909	3.31	68.294	85.290	217.463	234.083	2.16	−1.50
*qLA2_8*	*qLA1_8b, qLA3_8*	LA2	8	M8_2389	57.715	171.912	4.86	55.855	66.649	170.455	176.434	2.93	1.89
*qLA2_10*	*qLA1_10, qLA3_10*	LA2	10	M10_1121	33.420	125.573	4.28	30.773	38.624	110.756	134.519	2.49	−1.75
*qLA3_1a*	*qLA1_1a, qLA2_1a*	LA3	1	M1_1166	32.795	48.337	3.81	29.787	38.686	42.457	62.466	5.87	1.74
*qLA3_1b*	*qLA1_1b, qLA2_1b*	LA3	1	M1_2435	61.082	198.272	9.24	59.686	63.862	197.395	201.738	7.55	2.77
*qLA3_2a*		LA3	2	M2_129	4.558	2.802	5.91	0.385	5.830	1.334	3.419	11.74	4.03
*qLA3_2b*	*qLA1_2b, qLA2_2b*	LA3	2	M2_1194	41.982	56.798	10.86	39.663	45.227	48.215	103.717	11.20	4.06
*qLA3_2c*	*qLA1_2c*	LA3	2	M2_3360	81.294	239.246	3.97	78.860	89.575	235.987	240.608	3.81	1.83
*qLA3_3a*	*qLA2_3a*	LA3	3	M3_1500	35.920	160.130	8.98	34.654	38.227	156.318	168.871	6.80	−2.82
*qLA3_3b*	*qLA1_3b, qLA2_3b*	LA3	3	M3_2764	72.450	221.909	3.43	66.906	84.022	215.395	233.055	1.62	−1.43
*qLA3_8*	*qLA1_8b, qLA2_8*	LA3	8	M8_2444	60.836	174.152	3.74	55.855	69.779	170.455	177.712	2.79	1.90
*qLA3_10*	*qLA1_10, qLA2_10*	LA3	10	M10_1199	36.421	132.104	3.42	30.773	43.697	110.756	138.708	1.36	−1.35
*qFLA_2a*	*qLA1_2a, qLA2_2a, qTBA_2a*	FLA	2	M2_235	8.021	4.280	24.10	7.328	9.181	4.157	4.643	21.08	5.67
*qFLA_2b*		FLA	2	M2_2116	54.368	192.027	5.10	50.759	56.454	187.006	194.259	4.35	2.50
*qFLA_4*		FLA	4	M4_2580	66.584	241.604	3.77	62.652	70.067	238.274	242.881	3.11	2.03
*qFLA_5a*		FLA	5	M5_3058	62.087	202.984	6.07	56.661	63.813	182.116	210.045	2.65	3.01
*qFLA_5b*		FLA	5	M5_3747	82.921	219.988	11.52	80.145	85.962	219.017	220.891	8.75	−3.78
*qFLA_8*		FLA	8	M8_281	14.880	9.191	5.26	12.659	19.438	7.207	12.515	4.39	2.56
*qTBA_1a*		TBA	1	M1_764	22.958	30.613	3.38	18.091	30.017	19.586	43.581	0.85	3.35
*qTBA_1b*		TBA	1	M1_2052	49.527	160.088	9.26	48.607	54.022	117.695	182.705	8.83	−6.86
*qTBA_1c*		TBA	1	M1_3954	90.487	280.610	3.36	88.409	95.226	277.727	288.355	1.86	−3.17
*qTBA_2a*	*qLA1_2a, qLA2_2a, qFLA_2a*	TBA	2	M2_249	9.411	4.808	12.83	8.601	11.393	4.606	5.715	14.23	7.57
*qTBA_2b*		TBA	2	M2_1806	48.338	174.019	12.90	45.227	49.607	103.988	183.652	8.93	6.48
*qTBA_3a*		TBA	3	M3_443	23.480	16.956	6.00	20.086	24.979	11.691	20.883	6.95	4.91
*qTBA_3b*		TBA	3	M3_2361	57.633	203.076	12.56	56.253	58.668	198.496	206.503	9.25	6.70

^a^ Phenotypic variance explained. ^b^ Additive effects for detected QTLs.

**Table 3:** Summary of the overlap between QTLs controlling different angles. Upper right, the number of QTLs overlapping between two traits. Lower left, the percentage of variance explained by overlapping QTLs. The number to the left of the slash is variance explained for the trait in the same row and the number to the right of the slash is for the trait in the same column.

	LA1	LA2	LA3	FLA	TBA
LA1	NA	7	7	1	1
LA2	48.02/48.32	NA	7	1	1
LA3	34.21/38.70	37.19/42.97	NA	0	0
FLA	21.08/12.11	21.08/14.18	0	NA	1
TBA	14.23/12.11	14.23/14.18	0	14.23/21.08	NA

## Data Availability

All data needed to evaluate the results in the paper are present in the paper or the supplementary materials.

## Supplementary Materials

The following supporting information can be downloaded at: https://www.mdpi.com/article/10.3390/plants12030680/s1, Supplementary Table S1. The BLUP values and means in each environment for LA1, LA2, LA3, FLA and TBA of the DH lines.
